# Aquaporin‐4 knockout mice exhibit increased hypnotic susceptibility to ketamine

**DOI:** 10.1002/brb3.990

**Published:** 2018-05-09

**Authors:** Yunluo Lv, Wangshu Dai, Ai Ge, Yi Fan, Gang Hu, Yinming Zeng

**Affiliations:** ^1^ Department of Anesthesiology Nanjing First Hospital Nanjing Medical University Nanjing China; ^2^ The Comprehensive Cancer Centre of Drum Tower Hospital Medical School of Nanjing University Clinical Cancer Institute of Nanjing University Nanjing China; ^3^ Department of Respiratory Medicine Tongji Hospital Tongji University School of Medicine Shanghai China; ^4^ Jiangsu Key Laboratory of Neurodegeneration Department of Pharmacology Nanjing Medical University Nanjing China; ^5^ Jiangsu Province Institute of Anesthesiology Xuzhou Medical University Xuzhou China

**Keywords:** aquaporin‐4, ketamine, loss of the righting reflex, microdialysis, synaptic transmission

## Abstract

**Purpose:**

This study examines anesthetic/hypnotic effects of ketamine in AQP4 knockout (KO) and wild‐type (WT) mice with the particular focus on neurotransmission.

**Materials and Methods:**

Ketamine (100 mg/kg) was intraperitoneally injected in 16 WT and 16 KO mice. The hypnotic potencies were evaluated by the loss of the righting reflex (LORR). The amino acids neurotransmitter levels in prefrontal cortex were measured by microdialysis.

**Results:**

This study demonstrated that AQP4 knockout significantly shortened the latency compared with WT mice (98.0 ± 4.2 vs. 138.1 ± 15.0 s, *p* < .05) and prolonged duration of LORR (884.7 ± 58.6 vs. 562.0 ± 51.7 s, *p* < .05) compared with WT mice in LORR induced by ketamine. Microdialysis showed that lack of AQP4 markedly decreased glutamate level within 20 min (*p* < .05) and increased γ‐aminobutyric acid (GABA) level within 30–40 min (*p* < .05) after use of ketamine. Moreover, the levels of taurine were remarkably higher in KO mice than in WT mice, but no obvious differences in aspartate were observed between two genotypes.

**Conclusion:**

AQP4 deficiency led to more susceptibility of mice to ketamine, which is probably due to the modulation of specific neurotransmitters, hinting an essential maintenance of synaptic activity mediated by AQP4 in the action of ketamine.

## 1 INTRODUCTION

General anesthesia is a medically induced state of unconsciousness with loss of protective reflexes and alternations of neurotransmitters, resulting from the administration of one or more general anesthetic agents (Jevtovic‐Todorovic, 2016). Ketamine, a phencyclidine derivative agent, is widely used as a short‐acting “dissociative” general anesthetic (Li & Vlisides, 2016). Although ketamine is classically considered a noncompetitive N‐methyl‐D‐aspartate (NMDA) receptor antagonist, it is actually a wide‐ranging pleiotropic molecule that affects a variety of receptors and cellular processes. Ketamine blocks nicotinic acetylcholine ion channels, increases dopaminergic and noradrenergic neuromodulation, and it also acts as a weak agonist of delta and μ‐opioid receptors (Ivan Ezquerra‐Romano, Lawn, Krupitsky, & Morgan, 2018).

Glia express a large spectrum of neurotransmitter receptors and ionic channels to sense neuronal signals. Also, they sense and respond to neuronal activity via sophisticated calcium and sodium signaling, which are considered to be involved in the modulation of anesthetic sensitivity (Liu et al., 2016). As an attractive isoform with the features distinct from other aquaporins in central nervous system (CNS), aquaporin‐4 (AQP4) is predominantly expressed in glia, especially concentrated in the astrocyte endfeet membranes adjacent to the synaptic cleft and blood vessels (Miller, Moran, & Hall, 2016). AQP4 appears to be involved in the maintenance of cerebral homeostasis and serves as a modulator of astrocytic function (Amiry‐Moghaddam, Frydenlund, & Ottersen, 2004; Manley, Binder, Papadopoulos, & Verkman, 2004; Verkman, Binder, Bloch, Auguste, & Papadopoulos, 2006). Astrocytes are crucial elements implicated in neuronal activity and express receptors for most neuroactive compounds. As the importance of astrocytes in neuronal activity, AQP4 has gained sufficient attraction for its roles in the regulation of astrocytic function, such as astroglial migration (Saadoun et al., 2005) and activation(Fan et al., 2008), K^+^ buffering (Padmawar, Yao, Bloch, Manley, & Verkman, 2005), as well as neurotransmission (Ding et al., 2007; Fan et al., 2005; Sun et al., 2007).

Besides the contribution to support neurons, astrocytes are active participants in neuroregulation, including synaptic plasticity, neuronal communication, and neurosecretion (Chung, Allen, & Eroglu, 2015). It has been extensively demonstrated that astrocytes could “listen and talk” to synapses and mediate synaptic cross talk (Haydon & Carmignoto, 2006). Glutamate, an excitatory neurotransmitter in CNS, possesses powerful neurotoxin, and its accumulation may be implicated in the pathogenesis events such as amyotrophic lateral sclerosis and Alzheimer's disease (Karki, Smith, Johnson, Aschner, & Lee, 2015). γ‐Aminobutyric acid (GABA), an inhibitory neurotransmitter known to activate high‐affinity GABA receptor, elicits tonic inhibition to maintain a persistently suppressed tone (Yoon & Lee, 2014). Astrocytes participate in retrieving neurotransmitters released from active neurons, in particular glutamate and GABA (Colangelo, Cirillo, Lavitrano, Alberghina, & Papa, 2012), maintaining an appropriate environment for neuronal activity. Neurotransmitter‐gated ion channels are reported to be particularly sensitive to anesthetic agents, and general anesthetic action can be induced by potentiating inhibitory neurotransmission and/or weakening excitatory neurotransmission (Belelli, Lambert, Peters, Wafford, & Whiting, 1997; Grasshoff, Rudolph, & Antkowiak, 2005). It has been reported that GABA‐induced chloride transport potentiates anesthetic response mediated by anesthetic such as 1‐aminoanthracene (Butts et al., 2009). In terms of the important role of AQP4 in the modulation of synaptic activity and neurotransmission, we hypothesized that AQP4 might be responsible for the modulation of general anesthesia. In this study, an AQP4 knockout (KO) mouse model was used to decipher the potential effects of AQP4 on general anesthesia by measuring loss of the righting reflex (LORR) and the levels of several amino acid‐type neurotransmitters in the prefrontal cortex (PFC) in mice treated with ketamine.

## 2 METHODS

16 AQP4 knockout (KO) CD1 mice (aged 12–14 weeks, weighed 20–30 g) were used as an experiment group, and 16 wild‐type (WT) CD1 mice (12–14 weeks, 20–30 g) were treated as a control group. No significant differences were found in weights between CD1 and AQP4 null mice (24.51 ± 1.611 vs. 25.59 ± 0.739 s, *p* = .5464). The AQP4 KO mice were generated as described elsewhere (Ding et al., 2007) and had more than 99.99% homogeneity with WT mice after a series of backcrosses. All experiments were performed on body weight‐matched male wild‐type and AQP4 KO mice. Four mice per cage were housed under a standardized 12‐h light/dark cycle, and water and food were available ad libitum. The temperature of the testing room was kept at 22°C ± 2°C, and experiments were conducted between 9:00 and 17:00 on a thermostat. The mice were used only once in all experiments. The research protocols were approved by the Institutional Animal Care and Use Committee, Nanjing Medical University, China.

### 2.1 Behavioral observations

The sensitivity to ketamine was evaluated by a rating scale as previously described (Petrenko, Tsujita, Kohno, Sakimura, & Baba, 2007; Petrenko et al., 2004). Ketamine (Hengrui Pharmaceuticals Co., Ltd, Jiangsu, China) was dissolved in 0.9% saline solution, and its effective doses were chosen according to previous reports (Petrenko et al., 2004). Each mouse was intraperitoneally (ip) injected with ketamine (100 mg/kg) and placed in a 2‐L glass beaker. At a 2‐min interval, the beaker was tilted to an angle of approximately 45° with a horizontal plane three times to gently place animals on their backs, and the ability of each mouse to right itself was measured as the anesthetic score according to the rating scale as described by Boast et al. (Boast, Pastor, Gerhardt, Hall, & Liebman, 1988), with minor modifications as follows: 0: a normal righting reflex; +1: the mouse righted itself within 2 s in all three trials (slightly impaired righting reflex); +2: the latency to righting ranged at 2–10 s in three trails (moderately or severely impaired righting reflex); and +3:>10 s with no righting within for all three trials (i.e., LORR). Total anesthetic score (TAS) was the sum of all scores recorded after use of ketamine. The duration of LORR was calculated as the time between the LORR (as measured as a score of +3) and the time at which mice regained the ability to right themselves (as measured as a score of +2).

### 2.2 The measurement of amino acids in the prefrontal cortex

Concentrations of amino acids in the PFC were measured by microdialysis according to the protocols as described elsewhere (Ding et al., 2007; Semba, Adachi, & Arai, 2005). In brief, five weight‐matched male WT and KO mice were anesthetized with chloral hydrate (350 mg/kg, i.p.) and placed in a stereotaxic apparatus. After the scalp was incised, the skull was cleaned, and two drill holes for the fixing screws were dug. The microdialysis‐guided cannula was implanted into the right prefrontal cortex at the coordinates anterior–posterior (AP) +2.3 mm, medial–lateral (ML) ‐0.6 mm, dorsal–ventral (DV) ‐1.2 mm according to the stereotaxic atlas (Paxinos & Franklin, 1997). Microdialysis studies were conducted 7 days later in Plexiglas test chambers (Bioanalytical System, Inc., West Lafayette, Ind., USA). A microdialysis probe (MD‐2200, Bioanalytical system, Inc., West Lafayette, Ind., USA) with an active length of 2 mm was inserted gently through the guide cannula. The animals were lightly anesthetized with ether to facilitate manual insertion of the microdialysis probe into the guide cannula. The membranes were tested on the day before use.

On the day of testing, the microdialysis probe was perfused with an artificial cerebrospinal fluid (aCSF; 140 mM NaCl, 2.7 mM KCl, 1.4 mM CaCl_2_, 1.2 mM MgCl_2_, 5.0 mM glucose, pH 7.4) at a flow rate of 2 μl/min and lasted for 2 hr to reach an equilibrium state. After that, baseline samples were collected into polypropylene microcentrifuge vials at a 20‐min interval for 80 min. Following baseline sample collection, mice were injected with ketamine (100 mg/kg, i.p.), and dialysates were collected every 10 min for 200 min. In all, 20 μl of the dialysate samples was analyzed by high‐performance liquid chromatography (HPLC) with fluorescent detection (Zeng et al., 2007). The peak areas were integrated and calculated by means of an external standard calibration.

### 2.3 Statistical analysis

All values were presented as mean ± *SEM*. Statistical analysis for anesthetic sensitivity was performed with Student′s *t* test by SPSS version 20 (SPSS Inc., Chicago, IL, USA). One‐way ANOVA was used to analyze the differences in amino acids in the prefrontal cortex. *p* < .05 was considered statistically significant.

## 3 RESULTS

### 3.1 Effects of AQP4 knockout on hypnotic state induced by ketamine

**Figure 1:**
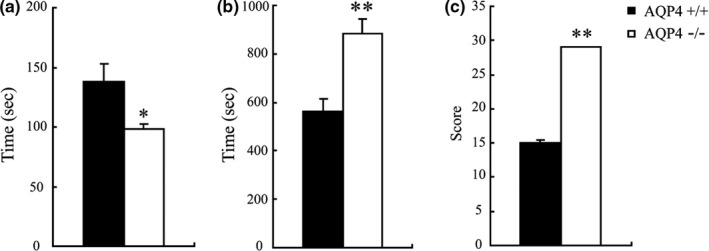
Latency of loss of righting reflex (a), duration of loss of righting reflex (b), and (c) total anesthetic scores (TASs) after intraperitoneal administration of ketamine (100 mg/kg) to WT and KO mice. The data were presented as mean ± *SEM*. *p < .05, **p < .01, WT vs KO mice

The LORR assay was conducted to determine whether AQP4 knockout could change the anesthetic/hypnotic responses to ketamine (100 mg/kg, i.p.). As shown in Figure 1, the latency of LORR (98.0 ± 4.2 s) was shorter in KO mice than in WT mice (138.1 ± 15.0 s, *p* < .05). Ketamine significantly prolonged the duration of LORR in KO mice compared with WT mice (884.7 ± 58.6 *vs*. 562.0 ± 51.7 s, *p* < .01). Furthermore, KO mice exhibited a twofold higher TAS than WT mice (29 ± 0.1 *vs*. 15 ± 0.4 s, *p* < .01) (Figure 1c). These findings suggested that AQP4 might be involved in anesthesia/hypnosis initiated by ketamine.

### 3.2 Effects of AQP4 deficiency on neurotransmitter release after use of ketamine

**Figure 2:**
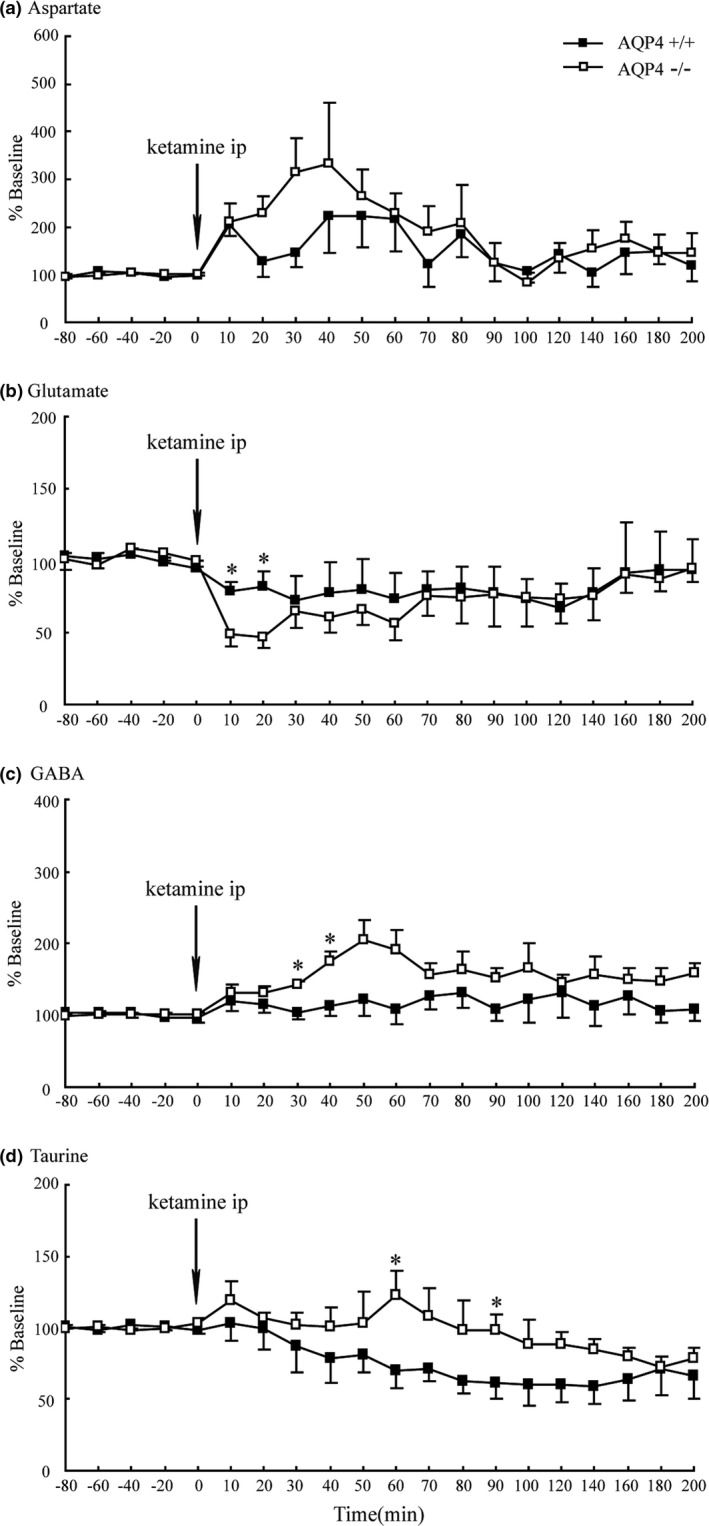
Effects of AQP4 deficiency on the levels of aspartate (a), glutamate (b), GABA (c), and taurine (d) in mouse prefrontal cortex after intraperitoneal administration of ketamine. Values are mean ± *SEM*. *p < .05 AQP4 WT vs KO mice

To evaluate changes in the release of neurotransmitters after use of ketamine, microdialysis was used to measure the levels of amino acids in the prefrontal cortex. Baseline levels of amino acids in WT and KO mice are summarized in Table 1. The levels of taurine were significantly increased in KO group compared with WT group (0.511 ± 0.004 *vs*. 0.425 ± 0.004 μM, *p* < .05). However, there were no significant differences in the basal levels of aspartate, glutamate, and GABA between WT and KO mice. As shown in Figure 2, the levels of amino acids in the prefrontal cortex changed rapidly in the two groups following ketamine administration. In addition to no statistical difference in the levels of aspartate between KO and WT mice (Figure 2a), the levels of glutamate were decreased significantly in KO mice within the first 20 min (Figure 2b). Moreover, the levels of GABA were increased notably in KO mice during 30–40 min after ketamine treatment (Figure 2c). And the levels of taurine were also remarkably elevated in KO mice at 60 and 90 min (Figure 2d). After that, the levels of aspartate, glutamate, GABA, and taurine were slowly recovered to their basal values, respectively.

**Table 1:** Basal values of dialysate neurotransmitters of amino acids in mouse prefrontal cortex (*n* = 5)

Animals	Aspartate (μM)	Glutamate (μM)	GABA (nM)	Taurine (μM)
WT	0.084 ± 0.002	0.194 ± 0.004	2.655 ± 0.055	0.425 ± 0.004
KO	0.063 ± 0.001	0.223 ± 0.011	2.881 ± 0.053	0.511 ± 0.004^a^

Values are mean ± *SEM*.

a *p *< .05 AQP4 WT vs KO mice.

## 4 DISCUSSION

General anesthesia is a reversible, drug‐interfered loss of consciousness. Accumulating evidence has demonstrated that AQP4 knockout or down‐regulation leads to increased seizure duration associated with aberrant neuronal activity (Binder, Yao, Zador, et al., 2006), suggesting an implication of AQP4 in the altered brain excitability and synaptic plasticity. As little is known about the role of AQP4 in anesthetic/hypnotic action of ketamine, AQP4 knockout mice were used to illustrate the function of AQP4 in hypnotic state. Our findings demonstrated that AQP4 knockout mice exhibited reduced latency and increased duration of LORR after use of ketamine, suggesting that AQP4 plays an essential role in the susceptibility of mice to ketamine.

AQP4, a predominant isoform of aquaporins in adult brain that is abundantly expressed in astrocytes’ endfeet, participates in the modulation of synaptic signals and the buffering of extracellular K^+^(Benarroch, 2005). As demonstrated previously by our laboratory in vitro and in vivo*,* AQP4 was implicated in cerebral neurotransmission (Ding et al., 2007; Zeng et al., 2007). It is well known that glutamate is the principal excitatory neurotransmitter released by presynaptic vesicular efflux and uptake by transporters (Halassa, Fellin, & Haydon, 2007; Semba et al., 2005) and that glutamate is a vital element in many physiological processes, such as the plasticity of synaptic transmission and the formation of neural networks (Matute, Domercq, & Sanchez‐Gomez, 2006). The disturbance of glutamate level could cause cognition and psychiatric illnesses, such as Alzheimer's disease, Parkinson's disease, epilepsy, and schizophrenia (Karki et al., 2015; Plaitakis et al., 2010; Rucker & McGuffin, 2014). Moreover, the removal of glutamate depends on the high‐affinity, sodium‐dependent glutamate transporter 1 (GLT‐1) on astrocytes, which promotes glutamate uptake from the extracellular space to maintain extracellular glutamate concentrations. AQP4 deficiency down‐regulates the expression of GLT‐1 on astrocytes and increases glutamate in the prefrontal cortex (Matute et al., 2006; Zeng et al., 2007). Thus, it is inferred that the susceptibility of mice to ketamine may be influenced by knockout of AQP4 due to the alteration in the levels of glutamate in synaptic cleft. Interestingly, using AQP4 knockout mice, we demonstrated that there was no significant difference in the basal values of glutamate between two genotypes, but that the levels of glutamate markedly decreased in KO mice compared with WT mice after use of ketamine. That seems not to be in accordance with the observations of others that AQP4 deficiency down‐regulates the expression of GLT‐1 and reduces glutamate uptake (Li et al., 2012; Zeng et al., 2007). In some respects, in vitro experiments are impossible to compare with in vivo experiments. Intricate mechanisms are implicated in AQP4, anesthetic state, and glutamate transport. Further explorations are needed to explain the phenomenon.

Ketamine, a phencyclidine derivative agent, has been widely used as an anesthetic that produces a wide spectrum of pharmacological effects, such as sedation, catalepsy, and somatic analgesia (Lodge & Mercier, 2015). It is found that ketamine is associated with neurocognitive performance, such as depression and schizophrenia (Dawson, Morris, & Pratt, 2013). Clinical trials have revealed that ketamine serves as an antidepressant at low doses, but that ketamine can evoke psychotomimetic actions at higher doses (Miller et al., 2016; Salvadore et al., 2012). In addition, ketamine is also an antagonist of the receptors of the N‐methyl‐D‐aspartate (NMDA) family, an ionotropic, ligand‐gated, glutamate‐sensitive neurotransmitter receptor (Lodge & Mercier, 2015). As general anesthetic can benefit the patient through suppressing of excitatory neurotransmission (Jevtovic‐Todorovic, 2016), our findings suggested that increased susceptibility to ketamine in KO mice might be ascribed to the lower levels of glutamate in the prefrontal cortex.

As an inhibitory neurotransmitter, GABA is released mainly from three candidate pathways: synaptic vesicle, GABA transporters, and non‐vesicular channel‐mediated release (Yoon & Lee, 2014). In particular, GABA transporters in glia promote the release of GABA into extracellular space, and subsequently GABA activates GABA_A_ receptors (Del, Bustamante, Del, & Solis, 2000; Yoon & Lee, 2014). GABA causes a long‐lasting and synchronous inhibition of mitral and granule cells in the olfactory bulb as well as tonic inhibition of neurons in the cerebellum, olfactory bulb, and hippocampus (Yoon & Lee, 2014). The previous studies have demonstrated that AQP4 deletion delays the clearance of K^+^ from the extracellular space, leading to an accumulation of extracellular K^+^ accompanied by persistent depolarization, which induces the release of massive neurotransmitters release (Strohschein et al., 2011). Our study also indicated that the levels of GABA rose rapidly in KO mice after use of ketamine, which may elicit a longer duration of LORR. Previous studies have revealed that GABA can potentiate response to anesthetic, such as 1‐aminoanthracene (Butts et al., 2009), indicating the important contribution of GABA to general anesthetic. Taken together with these coherent findings, AQP4 deficiency may enhance the hypnotic susceptibility to ketamine, which at least partially by enhanced high‐K^+^‐induced inhibitory neurotransmission. In addition, the synthesis and release of another endogenous agonist of GABA_A_ receptors (Martinez‐Torres & Miledi, 2004; Olson & Li, 2000), taurine, an essential neurotransmitter, may affect synaptic activity. The synthetic process of them depends on the cross talk among different cells from astrocytes to neurons, and the release event is sensitive to osmotic swelling and cellular volume (Strohschein et al., 2011). As previously reported by Ding et al., combining with our findings, the extracellular levels of taurine in KO mice were significant higher than those in WT mice (Fan et al., 2008). As AQP4 is involved in the regulation of cellular volume and osmotic homeostasis, we supposed that AQP4 deficiency induced the higher levels of taurine, which was responsible for increased hypnotic effect of ketamine.

## 5 CONCLUSIONS

In summary, this study suggested that AQP4 knockout could prolong the duration and shorten the latency of LORR, and increase TAS after use of ketamine, and that lack of AQP4 altered the neurotransmission of glutamate, GABA, and taurine. As astrocyte pathology and neurotransmission disturbance are important factors that devote to the pharmacodynamic actions of general anesthesia, our findings provide direct evidence that AQP4 plays a vital role in the anesthesia/hypnosis effect of ketamine, which is probably due to the altered neurotransmission, highlighting the important contribution of astrocytes in modulating drug‐induced general anesthesia.

## CONFLICT OF INTERESTS

None.

## AUTHOR CONTRIBUTION

Yunluo Lv: Conception and design of the study, analysis of data, manuscript writing. Wangshu Dai: Analysis of data, manuscript writing. Ai Ge: Conception of the study, acquisition of data. Yi Fan: Statistical analysis. Gang Hu: Design of the study. Yinming Zeng: Critical revision.

## References

[brb3990-bib-0001] Amiry‐Moghaddam, M. , Frydenlund, D. S. , & Ottersen, O. P. (2004). Anchoring of aquaporin‐4 in brain: Molecular mechanisms and implications for the physiology and pathophysiology of water transport. Neuroscience, 129(4), 999–1010. https://doi.org/10.1016/j.neuroscience.2004.08.049 15561415 10.1016/j.neuroscience.2004.08.049

[brb3990-bib-0002] Belelli, D. , Lambert, J. J. , Peters, J. A. , Wafford, K. , & Whiting, P. J. (1997). The interaction of the general anesthetic etomidate with the gamma‐aminobutyric acid type A receptor is influenced by a single amino acid. Proceedings of the National Academy of Sciences of the United States of America, 94(20), 11031–11036. https://doi.org/10.1073/pnas.94.20.11031 9380754 10.1073/pnas.94.20.11031 PMC23576

[brb3990-bib-0003] Benarroch, E. E. (2005). Neuron‐astrocyte interactions: Partnership for normal function and disease in the central nervous system. Mayo Clinic Proceedings, 80(10), 1326–1338. https://doi.org/10.4065/80.10.1326 16212146 10.4065/80.10.1326

[brb3990-bib-0005] Binder, D. K. , Yao, X. , Zador, Z. , Sick, T. J. , Verkman, A. S. , & Manley, G. T. (2006). Increased seizure duration and slowed potassium kinetics in mice lacking aquaporin‐4 water channels. Glia, 53(6), 631–636. https://doi.org/10.1002/glia.20318 16470808 10.1002/glia.20318

[brb3990-bib-0006] Boast, C. A. , Pastor, G. , Gerhardt, S. C. , Hall, N. R. , & Liebman, J. M. (1988). Behavioral tolerance and sensitization to CGS 19755, a competitive N‐methyl‐D‐aspartate receptor antagonist. Journal of Pharmacology and Experimental Therapeutics, 247(2), 556–561. 2846824

[brb3990-bib-0007] Butts, C. A. , Xi, J. , Brannigan, G. , Saad, A. A. , Venkatachalan, S. P. , Pearce, R. A. , & Dmochowski, I. J. (2009). Identification of a fluorescent general anesthetic, 1‐aminoanthracene. Proceedings of the National Academy of Sciences of the United States of America, 106(16), 6501–6506. https://doi.org/10.1073/pnas.0810590106 19346473 10.1073/pnas.0810590106 PMC2672486

[brb3990-bib-0008] Chung, W. S. , Allen, N. J. , & Eroglu, C. (2015). Astrocytes control synapse formation, function, and elimination. Cold Spring Harbor Perspectives in Biology, 7(9), a020370 https://doi.org/10.1101/cshperspect.a020370 25663667 10.1101/cshperspect.a020370 PMC4527946

[brb3990-bib-0009] Colangelo, A. M. , Cirillo, G. , Lavitrano, M. L. , Alberghina, L. , & Papa, M. (2012). Targeting reactive astrogliosis by novel biotechnological strategies. Biotechnology Advances, 30(1), 261–271. https://doi.org/10.1016/j.biotechadv.2011.06.016 21763415 10.1016/j.biotechadv.2011.06.016

[brb3990-bib-0010] Dawson, N. , Morris, B. J. , & Pratt, J. A. (2013). Subanaesthetic ketamine treatment alters prefrontal cortex connectivity with thalamus and ascending subcortical systems. Schizophrenia Bulletin, 39(2), 366–377. https://doi.org/10.1093/schbul/sbr144 22114100 10.1093/schbul/sbr144 PMC3576175

[brb3990-bib-0011] Del, O. N. , Bustamante, J. , Del, R. R. M. , & Solis, J. M. (2000). Taurine activates GABA(A) but not GABA(B) receptors in rat hippocampal CA1 area. Brain Research, 864(2), 298–307. 10802037 10.1016/s0006-8993(00)02211-3

[brb3990-bib-0012] Ding, J. H. , Sha, L. L. , Chang, J. , Zhou, X. Q. , Fan, Y. , & Hu, G. (2007). Alterations of striatal neurotransmitter release in aquaporin‐4 deficient mice: An in vivo microdialysis study. Neuroscience Letters, 422(3), 175–180. https://doi.org/10.1016/j.neulet.2007.06.018 17611025 10.1016/j.neulet.2007.06.018

[brb3990-bib-0013] Fan, Y. , Kong, H. , Shi, X. , Sun, X. , Ding, J. , Wu, J. , & Hu, G. (2008). Hypersensitivity of aquaporin 4‐deficient mice to 1‐methyl‐4‐phenyl‐1,2,3,6‐tetrahydropyrindine and astrocytic modulation. Neurobiology of Aging, 29(8), 1226–1236. https://doi.org/10.1016/j.neurobiolaging.2007.02.015 17353068 10.1016/j.neurobiolaging.2007.02.015

[brb3990-bib-0014] Fan, Y. , Zhang, J. , Sun, X. L. , Gao, L. , Zeng, X. N. , Ding, J. H. , & Hu, G. (2005). Sex‐ and region‐specific alterations of basal amino acid and monoamine metabolism in the brain of aquaporin‐4 knockout mice. Journal of Neuroscience Research, 82(4), 458–464. https://doi.org/10.1002/jnr.20664 16237719 10.1002/jnr.20664

[brb3990-bib-0015] Grasshoff, C. , Rudolph, U. , & Antkowiak, B. (2005). Molecular and systemic mechanisms of general anaesthesia: The ‘multi‐site and multiple mechanisms’ concept. Current Opinion in Anaesthesiology, 18(4), 386–391. https://doi.org/10.1097/01.aco.0000174961.90135.dc 16534263 10.1097/01.aco.0000174961.90135.dc

[brb3990-bib-0016] Halassa, M. M. , Fellin, T. , & Haydon, P. G. (2007). The tripartite synapse: Roles for gliotransmission in health and disease. Trends in Molecular Medicine, 13(2), 54–63. https://doi.org/10.1016/j.molmed.2006.12.005 17207662 10.1016/j.molmed.2006.12.005

[brb3990-bib-0017] Haydon, P. G. , & Carmignoto, G. (2006). Astrocyte control of synaptic transmission and neurovascular coupling. Physiological Reviews, 86(3), 1009–1031. https://doi.org/10.1152/physrev.00049.2005 16816144 10.1152/physrev.00049.2005

[brb3990-bib-0018] Ivan Ezquerra‐Romano, I. , Lawn, W. , Krupitsky, E. , & Morgan, C. J. A. (2018). Ketamine for the treatment of addiction: Evidence and potential mechanisms. Neuropharmacology, https://doi.org/10.1016/j.neuropharm.2018.01.017 10.1016/j.neuropharm.2018.01.017 29339294

[brb3990-bib-0019] Jevtovic‐Todorovic, V. (2016). General anesthetics and neurotoxicity: How much do we know? Anesthesiology Clinics, 34(3), 439–451. https://doi.org/10.1016/j.anclin.2016.04.001 27521190 10.1016/j.anclin.2016.04.001 PMC5477636

[brb3990-bib-0020] Karki, P. , Smith, K. , Johnson, J. Jr , Aschner, M. , & Lee, E. (2015). Role of transcription factor yin yang 1 in manganese‐induced reduction of astrocytic glutamate transporters: Putative mechanism for manganese‐induced neurotoxicity. Neurochemistry International, 88, 53–59. https://doi.org/10.1016/j.neuint.2014.08.002 25128239 10.1016/j.neuint.2014.08.002 PMC4326602

[brb3990-bib-0021] Li, L. , & Vlisides, P. E. (2016). Ketamine: 50 years of modulating the mind. Frontiers in Human Neuroscience, 10, 612 https://doi.org/10.3389/fnhum.2016.00612 27965560 10.3389/fnhum.2016.00612 PMC5126726

[brb3990-bib-0022] Li, Y. K. , Wang, F. , Wang, W. , Luo, Y. , Wu, P. F. , Xiao, J. L. , & Chen, J. G. (2012). Aquaporin‐4 deficiency impairs synaptic plasticity and associative fear memory in the lateral amygdala: Involvement of downregulation of glutamate transporter‐1 expression. Neuropsychopharmacology, 37(8), 1867–1878. https://doi.org/10.1038/npp.2012.34 22473056 10.1038/npp.2012.34 PMC3376319

[brb3990-bib-0023] Liu, X. , Gangoso, E. , Yi, C. , Jeanson, T. , Kandelman, S. , Mantz, J. , & Giaume, C. (2016). General anesthetics have differential inhibitory effects on gap junction channels and hemichannels in astrocytes and neurons. Glia, 64(4), 524–536. https://doi.org/10.1002/glia.22946 26666873 10.1002/glia.22946

[brb3990-bib-0024] Lodge, D. , & Mercier, M. S. (2015). Ketamine and phencyclidine: The good, the bad and the unexpected. British Journal of Pharmacology, 172(17), 4254–4276. https://doi.org/10.1111/bph.13222 26075331 10.1111/bph.13222 PMC4556466

[brb3990-bib-0025] Manley, G. T. , Binder, D. K. , Papadopoulos, M. C. , & Verkman, A. S. (2004). New insights into water transport and edema in the central nervous system from phenotype analysis of aquaporin‐4 null mice. Neuroscience, 129(4), 983–991. https://doi.org/10.1016/j.neuroscience.2004.06.088 15561413 10.1016/j.neuroscience.2004.06.088

[brb3990-bib-0026] Martinez‐Torres, A. , & Miledi, R. (2004). Expression of functional receptors by the human gamma‐aminobutyric acid A gamma 2 subunit. Proceedings of the National Academy of Sciences of the United States of America, 101(9), 3220–3223. https://doi.org/10.1073/pnas.0308682101 14981251 10.1073/pnas.0308682101 PMC365770

[brb3990-bib-0027] Matute, C. , Domercq, M. , & Sanchez‐Gomez, M. V. (2006). Glutamate‐mediated glial injury: Mechanisms and clinical importance. Glia, 53(2), 212–224. https://doi.org/10.1002/glia.20275 16206168 10.1002/glia.20275

[brb3990-bib-0028] Miller, O. H. , Moran, J. T. , & Hall, B. J. (2016). Two cellular hypotheses explaining the initiation of ketamine's antidepressant actions: Direct inhibition and disinhibition. Neuropharmacology, 100, 17–26. https://doi.org/10.1016/j.neuropharm.2015.07.028 26211972 10.1016/j.neuropharm.2015.07.028

[brb3990-bib-0029] Olson, J. E. , & Li, G. Z. (2000). Osmotic sensitivity of taurine release from hippocampal neuronal and glial cells. Advances in Experimental Medicine and Biology, 483, 213–218. https://doi.org/10.1007/0-306-46838-7_23 11787600 10.1007/0-306-46838-7_23

[brb3990-bib-0030] Padmawar, P. , Yao, X. , Bloch, O. , Manley, G. T. , & Verkman, A. S. (2005). K+ waves in brain cortex visualized using a long‐wavelength K + ‐sensing fluorescent indicator. Nature Methods, 2(11), 825–827. https://doi.org/10.1038/nmeth801 16278651 10.1038/nmeth801

[brb3990-bib-0031] Paxinos, G. , & Franklin, K. B. J. . (1997). The mouse brain in stereotaxic coordinates. Orlando: Academic Press.

[brb3990-bib-0032] Petrenko, A. B. , Tsujita, M. , Kohno, T. , Sakimura, K. , & Baba, H. (2007). Mutation of alpha1G T‐type calcium channels in mice does not change anesthetic requirements for loss of the righting reflex and minimum alveolar concentration but delays the onset of anesthetic induction. Anesthesiology, 106(6), 1177–1185. https://doi.org/10.1097/01.anes.0000267601.09764.e6 17525593 10.1097/01.anes.0000267601.09764.e6

[brb3990-bib-0033] Petrenko, A. B. , Yamakura, T. , Fujiwara, N. , Askalany, A. R. , Baba, H. , & Sakimura, K. (2004). Reduced sensitivity to ketamine and pentobarbital in mice lacking the N‐methyl‐D‐aspartate receptor GluRepsilon1 subunit. Anesthesia and Analgesia, 99(4), 1136–1140, table of contents. https://doi.org/10.1213/01.ane.0000131729.54986.30 15385364 10.1213/01.ANE.0000131729.54986.30

[brb3990-bib-0034] Plaitakis, A. , Latsoudis, H. , Kanavouras, K. , Ritz, B. , Bronstein, J. M. , Skoula, I. , & Spanaki, C. (2010). Gain‐of‐function variant in GLUD2 glutamate dehydrogenase modifies Parkinson's disease onset. European Journal of Human Genetics, 18(3), 336–341. https://doi.org/10.1038/ejhg.2009.179 19826450 10.1038/ejhg.2009.179 PMC2987208

[brb3990-bib-0035] Rucker, J. J. , & McGuffin, P. (2014). Chipping away at major depressive disorder. Genome Biology, 15(7), 421 https://doi.org/10.1186/s13059-014-0421-3 25315250 10.1186/s13059-014-0421-3 PMC4220094

[brb3990-bib-0036] Saadoun, S. , Papadopoulos, M. C. , Watanabe, H. , Yan, D. , Manley, G. T. , & Verkman, A. S. (2005). Involvement of aquaporin‐4 in astroglial cell migration and glial scar formation. Journal of Cell Science, 118(Pt 24), 5691–5698. https://doi.org/10.1242/jcs.02680 16303850 10.1242/jcs.02680

[brb3990-bib-0037] Salvadore, G. , van der Veen, J. W. , Zhang, Y. , Marenco, S. , Machado‐Vieira, R. , Baumann, J. , & Zarate, C. A. Jr (2012). An investigation of amino‐acid neurotransmitters as potential predictors of clinical improvement to ketamine in depression. International Journal of Neuropsychopharmacology, 15(8), 1063–1072. https://doi.org/10.1017/s1461145711001593 22040773 10.1017/S1461145711001593 PMC3342437

[brb3990-bib-0038] Semba, K. , Adachi, N. , & Arai, T. (2005). Facilitation of serotonergic activity and amnesia in rats caused by intravenous anesthetics. Anesthesiology, 102(3), 616–623. https://doi.org/10.1097/00000542-200503000-00021 15731601 10.1097/00000542-200503000-00021

[brb3990-bib-0039] Strohschein, C. , Huttmann, K. , Gabriel, S. , Binder, D. K. , Heinemann, U. , & Steinhauser, C. (2011). Impact of aquaporin‐4 channels on K+ buffering and gap junction coupling in the hippocampus. Glia, 59(6), 973–980. https://doi.org/10.1002/glia.21169 21446052 10.1002/glia.21169

[brb3990-bib-0040] Sun, X. L. , Ding, J. H. , Fan, Y. , Zhang, J. , Gao, L. , & Hu, G. (2007). Aquaporin 4 regulates the effects of ovarian hormones on monoamine neurotransmission. Biochemical and Biophysical Research Communications, 353(2), 457–462. https://doi.org/10.1016/j.bbrc.2006.12.040 17196551 10.1016/j.bbrc.2006.12.040

[brb3990-bib-0041] Verkman, A. S. , Binder, D. K. , Bloch, O. , Auguste, K. , & Papadopoulos, M. C. (2006). Three distinct roles of aquaporin‐4 in brain function revealed by knockout mice. Biochimica et Biophysica Acta, 1758(8), 1085–1093. https://doi.org/10.1016/j.bbamem.2006.02.018 16564496 10.1016/j.bbamem.2006.02.018

[brb3990-bib-0042] Yoon, B. E. , & Lee, C. J. (2014). GABA as a rising gliotransmitter. Frontiers in Neural Circuits, 8, 141 https://doi.org/10.3389/fncir.2014.00141 25565970 10.3389/fncir.2014.00141 PMC4269106

[brb3990-bib-0043] Zeng, X. N. , Sun, X. L. , Gao, L. , Fan, Y. , Ding, J. H. , & Hu, G. (2007). Aquaporin‐4 deficiency down‐regulates glutamate uptake and GLT‐1 expression in astrocytes. Molecular and Cellular Neurosciences, 34(1), 34–39. https://doi.org/10.1016/j.mcn.2006.09.008 17074507 10.1016/j.mcn.2006.09.008

